# Comparison of the Association Between Goal-Directed Planning and Self-reported Compulsivity vs Obsessive-Compulsive Disorder Diagnosis

**DOI:** 10.1001/jamapsychiatry.2019.2998

**Published:** 2019-10-09

**Authors:** Claire M. Gillan, Eyal Kalanthroff, Michael Evans, Hilary M. Weingarden, Ryan J. Jacoby, Marina Gershkovich, Ivar Snorrason, Raphael Campeas, Cynthia Cervoni, Nicholas Charles Crimarco, Yosef Sokol, Sarah L. Garnaat, Nicole C. R. McLaughlin, Elizabeth A. Phelps, Anthony Pinto, Christina L. Boisseau, Sabine Wilhelm, Nathaniel D. Daw, H. B. Simpson

**Affiliations:** 1 School of Psychology, Trinity College Institute of Neuroscience and Global Brain Health Institute, Trinity College Dublin, Dublin, Ireland; 2 Department of Psychology, The Hebrew University of Jerusalem, Mount Scopus, Israel; 3 Department of Psychology, New York University, New York; 4 Department of Psychiatry, Massachusetts General Hospital, Boston; 5 Department of Psychiatry, Harvard Medical School, Boston, Massachusetts; 6 Department of Psychiatry, Columbia Irving University Medical Center, New York, New York; 7 New York State Psychiatric Institute, New York; 8 Department of Psychiatry, Harvard Medical School, McLean Hospital, Belmont, Massachusetts; 9 Department of Psychiatry, Stony Brook University, Stony Brook, New York; 10 VISN 2 Mental Illness Research Education and Clinical Centers, New York, New York; 11 James J. Peters Veterans Affairs Medical Center, Bronx, New York; 12 Icahn School of Medicine at Mount Sinai, New York, New York; 13 Warren Alpert Medical School of Brown University, Providence, Rhode Island; 14 Butler Hospital, Providence, Rhode Island; 15 Department of Psychology, Harvard University, Cambridge, Massachusetts; 16 Department of Psychiatry, Hofstra Northwell School of Medicine, Hempstead, New York; 17 Department of Psychiatry and Behavioral Sciences, Northwestern University Feinberg School of Medicine, Chicago, Illinois; 18 Princeton Neuroscience Institute, Department of Psychology, Princeton University, Princeton, New Jersey

## Abstract

**Importance:**

Dimensional definitions of transdiagnostic mental health problems have been suggested as an alternative to categorical diagnoses, having the advantage of capturing heterogeneity within diagnostic categories and similarity across them and bridging more naturally psychological and neural substrates.

**Objective:**

To examine whether a self-reported compulsivity dimension has a stronger association with goal-directed and related higher-order cognitive deficits compared with a diagnosis of obsessive-compulsive disorder (OCD).

**Design, Setting, and Participants:**

In this cross-sectional study, patients with OCD and/or generalized anxiety disorder (GAD) from across the United States completed a telephone-based diagnostic interview by a trained rater, internet-based cognitive testing, and self-reported clinical assessments from October 8, 2015, to October 1, 2017. Follow-up data were collected to test for replicability.

**Main Outcomes and Measures:**

Performance was measured on a test of goal-directed planning and cognitive flexibility (Wisconsin Card Sorting Test [WCST]) and a test of abstract reasoning. Clinical variables included *DSM-5* diagnosis of OCD and GAD and 3 psychiatric symptom dimensions (general distress, compulsivity, and obsessionality) derived from a factor analysis.

**Results:**

Of 285 individuals in the analysis (mean [SD] age, 32 [12] years; age range, 18-77 years; 219 [76.8%] female), 111 had OCD; 82, GAD; and 92, OCD and GAD. A diagnosis of OCD was not associated with goal-directed performance compared with GAD at baseline (β [SE], −0.02 [0.02]; *P* = .18). In contrast, a compulsivity dimension was negatively associated with goal-directed performance (β [SE], −0.05 [0.02]; *P* = .003). Results for abstract reasoning task and WCST mirrored this pattern; the compulsivity dimension was associated with abstract reasoning (β [SE], 2.99 [0.63]; *P* < .001) and several indicators of WCST performance (eg, categories completed: β [SE], −0.57 [0.09]; *P* < .001), whereas OCD diagnosis was not (abstract reasoning: β [SE], 0.39 [0.66]; *P* = .56; categories completed: β [SE], −0.09 [0.10]; *P* = .38). Other symptom dimensions relevant to OCD, obsessionality, and general distress had no reliable association with goal-directed performance, WCST, or abstract reasoning. Obsessionality had a positive association with requiring more trials to reach the first category on the WCST at baseline (β [SE], 2.92 [1.39]; *P* = .04), and general distress was associated with impaired goal-directed performance at baseline (β [SE],−0.04 [0.02]; *P* = .01). However, unlike the key results of this study, neither survived correction for multiple comparisons or was replicated at follow-up testing.

**Conclusions and Relevance:**

Deficits in goal-directed planning in OCD may be more strongly associated with a compulsivity dimension than with OCD diagnosis. This result may have implications for research assessing the association between brain mechanisms and clinical manifestations and for understanding the structure of mental illness.

## Key Points

**Question:**

Are deficits in goal-directed planning better identified by self-reported compulsivity or a diagnosis of obsessive-compulsive disorder?

**Findings:**

In this cross-sectional study of 285 patients diagnosed with obsessive-compulsive disorder, generalized anxiety disorder, or both, self-reported compulsivity was more strongly associated with goal-directed deficits than a diagnosis of obsessive-compulsive disorder compared with generalized anxiety disorder.

**Meaning:**

This study suggests that transdiagnostic compulsivity symptoms may have greater biological validity than a diagnosis of obsessive-compulsive disorder.

## Teaser

This cross-sectional study assesses whether deficits in goal-directed planning are better identified by self-reported compulsivity or a diagnosis of obsessive-compulsive disorder.

## Introduction

Fundamental issues with the use of *DSM-5*^1^ and *International Classification of Mental and Behavioural Disorders, 10th Revision*^2^ disorder categories for neurobiological research are increasingly recognized.^3,4,5,6^ Diagnostic groups are highly heterogeneous; patients often have the same diagnosis with vastly different symptom profiles.^7^ Moreover, comorbidity is the rule rather than the exception.^8^ Individuals with no psychiatric diagnosis are usually the control group, even though they differ from patients with a diagnosis in many ways beyond the diagnosis under investigation, including anxiety,^9^ depression,^10^ physical illness,^11^ and early-life adversity.^12^ As a result, potential biomarkers, intermediate phenotypes, or etiologic substrates can at best show a modest association with a categorical clinical phenotype, and this association is unlikely to be specific to that phenotype.

Whether there is an alternative way to conceptualize psychiatric ill health that might provide a closer and more specific fit to underlying biological states remains unknown. A long-standing suggestion has been to dispense with categories and instead study graded clinical phenotypes (dimensions) that manifest transdiagnostically.^13^ Although initial results have been promising,^14,15,16,17^ whether a dimensional framework for understanding psychiatric states provides a better match to brain-based measures than the extant categorical one remains an open question.

We tested this question with respect to goal-directed control, a cognitive capacity that protects against forming maladaptive habits^18,19,20^ and has been suggested to underlie compulsivity in obsessive-compulsive disorder (OCD)^21,22,23,24,25,26^ and other compulsive disorders, such as addiction^27,28^ and binge-eating disorder.^29^ Goal-directed control refers to our ability to make prospective decisions, to simulate alternative futures, and to make decisions that align with our current needs and wants.^30^ It has well-defined neural substrates^25,30,31^ and pharmacologic correlates^32,33^ and has been computationally formalized^30^ and studied across species.^27^ Like almost all biomarkers in psychiatry, issues of specificity have emerged, with other disorders showing impairment in goal-directed control.^34,35,36^ Recent evidence from a large internet-based general population study^17^ found that a transdiagnostic dimension relating to compulsive behavior and intrusive thought might explain this pattern of nonspecific results. However, it is currently unknown whether these results from the general population apply to patients and, more importantly, how these self-reported dimensions compare with *DSM-5* categories.

The current study investigated self-reported dimensions vs disorder diagnoses using an internet-based, dimensional method to assess patients in whom diagnoses were established using a structured clinical telephone interview. Given the confounders associated with using a healthy control group with no diagnosis (eg, comorbid anxiety, depression, and life stress), we recruited individuals with generalized anxiety disorder (GAD) to serve as a patient control group. We selected this disorder because the clinical presentation of GAD does not involve compulsive behavior and individual differences in trait anxiety have not been linked to goal-directed planning deficits.^14,17^ However, GAD shares a pattern of excessive worry and intrusive thoughts with OCD as well as nonspecific clinical features, such as general distress and impairment.^1^ The use of patients with GAD allowed us to assess the potential contribution of these features to goal-directed deficits in OCD, separating compulsivity from obsessionality and general distress.

We hypothesized that goal-directed deficits in patients with diagnoses of OCD, GAD, or both would be specifically associated with a compulsivity dimension that manifests transdiagnostically and that this would outperform OCD diagnosis in its association with goal-directed deficits. We also probed the generality of our findings to other, related, aspects of higher-order cognition, which have been consistently linked to OCD diagnosis but not previously studied in the context of compulsivity. We investigated cognitive flexibility^37,38^ and abstract reasoning^39^ as a first step because, like goal-directed planning, these are executive functions that rely on the integrity of the prefrontal cortex and striatum^40,41^; thus, we reasoned that they represent good candidate characteristics of a compulsivity dimension.

## Methods

### Participants

In this cross-sectional study, 285 participants were recruited from across the United States using internet-based advertising. A total of 1136 individuals expressed an interest, of whom 394 met the inclusion criteria (likely diagnosis of OCD and/or GAD, English language, and no history of stroke, neurologic problems, or head injury) and responded to a scheduling email. These individuals were interviewed over the telephone using an electronic version of the Mini-International Neuropsychiatric Interview, version 7.0 for *DSM-5*. Patients completed all study components remotely from October 8, 2015, to October 1, 2017. A total of 335 individuals met the criteria for OCD or GAD, 43 did not, and an additional 16 withdrew. Of the 335 invited to participate, 285 completed the study. Participants were paid $20 plus a small bonus (<$3). Additional details of the recruitment and screening can be found in the eMethods in the Supplement. Ethical approval was obtained from the New York University Committee on Activities Involving Human Subjects, in addition to institutional review board approvals from the New York State Psychiatric Institute, Care New England–Butler Hospital, Massachusetts General Hospital Partners Human Research Committee, and Northwell Health. All participants provided electronic informed consent. Data were stored in a pseudo-anonymized format.

### Goal-Directed Control

**Figure 1.**
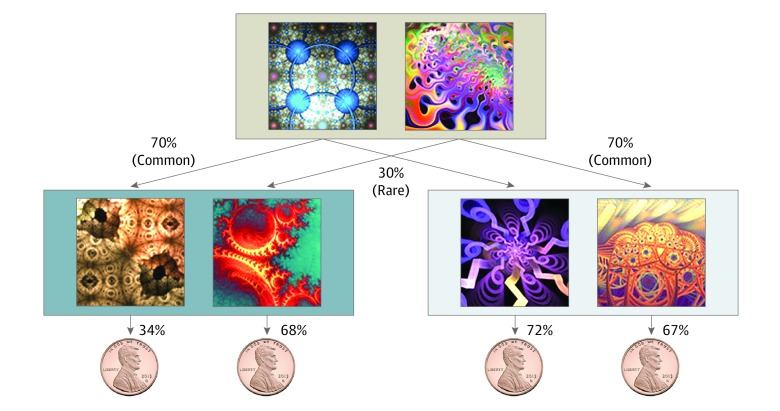
Goal-Directed Planning Task Goal-directed (model-based) planning was assessed using a 2-step decision-making task.^42^ In each trial, individuals were asked to select between 1 of 2 choices (top). On the basis of the depicted probabilities (70% or 30%) for each of these options, individuals would transition to a second stage, where they were again asked to choose between 2 options. These choices were rewarded (or not rewarded) with a 1-cent coin based on the current probability of reward assigned to that fractal. In the example trial depicted here, the leftmost fractal had a 34% chance of producing a coin. This probability changed slowly throughout the task, encouraging individuals to update their action preferences and regularly explore new options.

Participants completed a 2-step decision-making task that allowed us to derive individual estimates of model-based planning^42^ (Figure 1). Model-based planning is considered as a formalization of goal-directed control, such that choices are made prospectively and influenced by known environmental contingencies and the current desirability of rewards. Specifically, model-based planning reflects the extent to which individuals use their knowledge of the transition structure of the task to make choices. For example, if a first-stage choice is followed by a rare (30%) transition and individuals ultimately get a reward at stage 2, they should be less likely to repeat that choice on the next trial because the alternative choice has a higher probability (ie, common, 70%) of returning them to that valuable second-stage state. To measure goal-directed (model-based) control, we used a regression-based procedure^17^ documented in the eMethods in the Supplement. This procedure was complemented with computational modeling (eMethods in the Supplement).

### Other Cognitive Tests

Cognitive flexibility was assessed using the Wisconsin Card Sorting Test (WCST).^43^ Abstract reasoning ability was assessed using a task^17^ based on Raven’s Progressive Matrices^44^ (more information is available in the eMethods in the Supplement).

### Self-report Symptoms

Participants completed the Obsessive-Compulsive Inventory–Revised (OCI-R),^45^ which has 6 subscales (washing, checking, neutralizing, counting, hoarding, and obsessing); the Metacognitive Beliefs Questionnaire (MCQ^46^), which has 5 subscales (cognitive confidence, positive beliefs about worry, cognitive self-consciousness, negative beliefs about uncontrollability and danger, and need to control thoughts); the Depression and Anxiety and Stress Scale (DASS^47^), which has 3 subscales (depression, anxiety, and stress); and the Sheehan Disability Scale (SDS^48^), which assesses functional impairment arising from one’s disorder.

### Follow-up Testing

To assess reproducibility, we invited all individuals to participate again, and data were collected from 110 participants. Procedures were identical to testing session 1 except that we did not conduct the telephone interview again. Data were collected in a single wave with a mean (SD) of 413 (144) days since the initial assessment (range, 42-685 days).

### Statistical Analysis

Cognitive test performance was analyzed to produce individual participant’s scores (eMethods in the Supplement), which were brought forward to secondary analyses to test the association with clinical variables. The association of OCD diagnosis (independent variable) with cognition was determined using regression analysis in R, version 3.5.2 (lme4 package; R Foundation for Statistical Computing). Results from this analysis correspond to OCD and OCD plus GAD vs GAD. Likewise, analysis of the association of GAD with cognition corresponds to GAD and OCD plus GAD vs OCD. The analysis for dimensions was identical except that the independent variables were continuous. Because of the overlapping set of variables, we reported results from separate models for each psychiatric variable (ie, not controlling for one another). When significant associations were revealed for more than 1 variable, we addressed comparative questions using combined analyses to disambiguate the variable influencing the association. Independent variables in all analyses were zero centered. Significance was assessed using a 2-tailed *P*<.05 significance threshold, but for analysis of the 6 cognitive measures, we also assessed whether findings remained after a more stringent Bonferroni correction that corresponded to an adjusted α = .008. Supplementary analyses are presented in the eResults in the Supplement, including additional controls for age, medication, and comorbidity.

## Results

### Diagnosis

Analysis was conducted for 285 individuals (mean [SD] age, 32 [12] years; age range, 18-77 years; 219 [76.8%] female), 111 with OCD, 82 with GAD, and 92 with OCD and GAD. The sample was racially and ethnically representative.^49^ That is, 211 (74.0%) were white, 34 (11.9%) black, 19 (6.7%) Asian, 4 (1.4%) American Indian or Alaskan Native, 1 (0.4%) Native Hawaiian or other Pacific Islander, and 16 (5.6%) nondisclosed. In terms of ethnicity, 238 (83.5%) were non-Hispanic, 33 (11.6%) Latino/Hispanic, and 14 (4.9%) nondisclosed. Demographic and clinical characteristics of the sample are given in Table 1. A total of 157 individuals were receiving treatment (55.1%), and the proportion did not differ as a function of diagnosis (Table 1). A diagnosis of OCD was associated with higher scores on the OCI-R and the MCQ but not the DASS or SDS. Conversely, a GAD diagnosis was associated with lower OCI-R scores and higher DASS scores but had no association with MCQ or SDS scores. Individuals with an OCD diagnosis were older (mean [SD] age, 36.6 [12.9] years) than those without (mean [SD] age, 31.2 [11.4] years) (β [SE], 2.48 [0.74]; *P* < .001). As in a previous study,^17^ age was associated with goal-directed planning, with younger persons performing better (β [SE], −0.05 [0.02], *P* = .002). Thus, age was controlled for in all analyses. No significant association was found between OCD (β [SE], −0.02 [0.02]; *P* = .18) and GAD diagnoses (β [SE], −0.01 [0.02]; *P* = .71) and model-based planning (Table 1). Secondary analyses revealed similar results for cognitive flexibility and abstract reasoning. OCD diagnosis was not significantly associated with any of these measures (categories complete: β [SE], −0.09 [0.10]; *P* = .38; nonperseverative errors: β [SE], 1.37 [1.07]; *P* = .20; trials to first category: β [SE], 2.23 [1.41]; *P* = .12; perseverative errors: β [SE], −0.15 [0.59]; *P* = .80) on the WCST and with abstract reasoning (β [SE], 0.39 [0.66]; *P* = .56).

**Table 1.** Demographics, Clinical Characteristics, and Cognitive Task Performance by Diagnosis^a^

Variable	OCD (n = 111)	GAD (n = 82)	OCD and GAD (n = 92)	OCD Diagnosis			GAD Diagnosis	
				β (SE)	*P* Value	β (SE)		*P* Value
Demographics								
Age, y	37.50 (12.69)	31.16 (11.0)	35.59 (13.2)	2.48 (0.74)	<.001	−1.96 (0.75)		.009
Female, No. (%)	81 (73.0)	68 (82.9)	70 (76.1)	2.40^b^	.12	1.53^b^		.22
Medicated, No. (%)	58 (52.3)	43 (52.4)	56 (60.9)	0.33^b^	.57	0.59^b^		.44
Symptoms								
OCI-R	36.96 (16.49)	20.38 (13.62)	35.17 (15.73)	7.15 (0.92)	<.001	−4.28 (0.98)		<.001
DASS	50.67 (30.67)	55.32 (27.04)	67.72 (29.63)	1.40 (1.79)	.44	5.47 (1.76)		.002
MCQ	43.64 (17.78)	40.24 (17.04)	50.77 (16.51)	3.01 (1.03)	.003	1.06 (1.05)		.31
SDS	18.45 (8.32)	17.83 (7.32)	19.96 (7.59)	0.59 (0.46)	.20	0.25 (0.47)		.60
2-Step task^c^								
Model based	0.15 (0.24)	0.20 (0.29)	0.11 (0.27)	−0.02 (0.02)	.18	−0.01 (0.02)		.71
WCST^c^								
Categories completed	4.76 (1.89)	5.28 (1.57)	4.88 (1.78)	−0.09 (0.10)	.38	0.05 (0.10)		.60
Perseverative errors	11.70 (9.73)	11.27 (11.77)	12.27 (8.84)	−0.15 (0.59)	.80	0.42 (0.59)		.48
Nonperseverative errors	22.07 (21.79)	15.41 (14.39)	19.74 (17.71)	1.37 (1.07)	.20	−1.20 (1.06)		.26
Trials to first category	26.70 (28.80)	17.89 (15.08)	23.92 (24.12)	2.23 (1.41)	.12	−1.78 (1.40)		.21
Matrices test^c^								
Abstract reasoning	90.67 (11.67)	91.96 (11.33)	92.00 (11.31)	0.39 (0.66)	.56	0.10 (0.66)		.88

Abbreviations: DASS, Depression and Anxiety Severity Scale; GAD, generalized anxiety disorder; MCQ, Metacognitive Beliefs Questionnaires; OCD, obsessive-compulsive disorder; OCI-R, Obsessive-Compulsive Inventory–Revised; SDS, Sheehan Disability Scale; WCST, Wisconsin Card Sorting Test.

^a^ Data are reported as mean (SD) unless otherwise indicated.

^b^ χ^2^ Value.

^c^ Analysis controls for age.

### Dimensions

**Figure 2.**
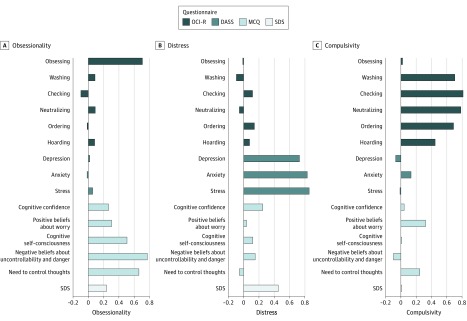
Factor Analysis of 3 Transdiagnostic Dimensions: Compulsivity, Obsessionality, and General Distress Each bar represents the loadings for each subscale onto the 3 factors (distress, compulsivity, and obsessionality). The height of each bar reflects its loading onto the relevant factor. Color codes indicate the questionnaire from which each subscale was drawn. DASS indicates Depression and Anxiety and Stress Scale; MCQ, Metacognitive Beliefs Questionnaire; OCI-R, Obsessive-Compulsive Inventory–Revised; SDS, Sheehan Disability Scale.

To achieve a better separation of obsessions from compulsions, we conducted a factor analysis using the subscales of the OCI-R, DASS, and MCQ and the global disability score from the SDS (Figure 2). The result was 3 factors (dimensions) that constituted a slight but informative reformulation. The SDS loaded with anxiety, depression, and stress from the DASS, comprising a general distress factor. Most critically, the obsessions subscale from the OCI-R loaded strongly with all subscales from the MCQ to form an obsessionality factor, whereas the other 5 of the 6 subscales on the OCI-R loaded together on a separate compulsivity factor. Results for the original questionnaires are presented in eTable 1 through eTable 5 in the Supplement. Similar to OCD diagnosis, the compulsivity factor was associated with older age (*r* = 0.17; *P* = .005). Obsessionality was associated with a younger age (*r* = −0.14; *P* = .02), and general distress showed no association (*r* = −0.09; *P* = .12). Patients receiving medication had lower scores on the compulsivity dimension (β [SE], −0.28 [0.11]; *P* = .02). Ancillary analyses of the association of age and treatment with the results are presented in eFigure 1 and eFigure 2 in the Supplement.

**Figure 3.**
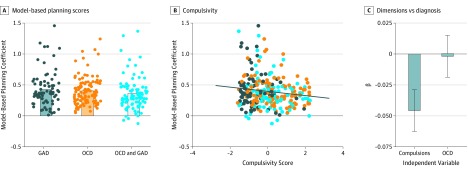
Association Among Model-Based Planning Scores, Diagnostic Status, and Compulsivity A, Bars display mean model-based planning scores (after controlling for age) by group, and dots indicate individual participant’s performance. No significant association was found for obsessive-compulsive disorder (OCD) (*P* = .18) or generalized anxiety disorder (GAD) diagnosis (*P* = .71) with model-based planning. The coefficients are from a regression model, specifically the interaction between reward and transition on stay behavior in the 2-step task. Scores below 0 were possible but rare (5 of 285 scores were below 0); scores close to 0 indicated a poor fit of the model to behavior. B, Scatterplot depicting the association between scores on the transdiagnostic compulsivity dimension and model-based planning ability, controlling for age. A significant negative association was found (*P* = .003). Individuals who had the highest self-reported compulsivity had the lowest scores on the test of model-based planning. Colors indicate the diagnoses for which each participant met the criteria (OCD, GAD, or combined OCD and GAD). C, Results are from a regression analysis comparing OCD diagnosis with the dimensional compulsivity factor in the same analysis. The association of OCD with model-based planning approached 0 when compulsivity was included in the same model (*P* = .91), whereas the association with compulsivity remained strong (*P* = .007). Error bars indicate SEs.

The compulsivity factor was associated with model-based planning failures (Figure 3A), but the obsessionality factor was not (Figure 3B and Table 2). A significant association was found between the general distress factor and model-based planning (β [SE], −0.04 [0.02]; *P* = .01), but the association was not maintained after Bonferroni correction for multiple comparisons, or after controlling for compulsivity in the uncorrected model (β [SE], −0.02 [0.02]; *P* = .19). Secondary results for other cognitive tests showed a similar pattern. In terms of cognitive flexibility (WCST), the compulsive factor was associated with fewer categories completed (β [SE], −0.57 [0.09]; *P* < .001) and more nonperseverative errors (β [SE], 5.86 [1.01]; *P* < .001) and trials to complete the first category (β [SE], 6.32 [1.36]; *P* < .001). Perseverative errors were not significantly associated with compulsivity (β [SE], 0.87 [0.59]; *P* = .14). In addition, no association was found between the obsessionality factor and WCST, with the exception of an increase in the number of trials to complete the first category (β [SE], 2.92 [1.39]; *P* = .04), but the association was not maintained after correction for multiple comparisons. The compulsivity factor was associated with deficits in abstract reasoning (β [SE], −2.99 [0.63]; *P* < .001), whereas general distress (β [SE], −1.02 [0.65]; *P* = .12) and obsessionality (β [SE], −0.42 [0.65]; *P* = .52) were not (Table 2).

**Table 2.** Cognitive Test Performance and Dimensions

Test	General Distress		Compulsivity			Obsessionality		
	β (SE)	*P* Value	β (SE)	*P* Value	β (SE)		*P* Value
2-Step task^a^							
Model based	−0.04 (0.02)	.01	−0.05 (0.02)	.003	−0.01 (0.02)		.63
WCST^a^							
Categories completed	−0.11 (0.10)	.26	−0.57 (0.09)	<.001	−0.06 (0.10)		.53
Perseverative errors	−0.02 (0.59)	.97	0.87 (0.59)	.14	−1.05 (0.59)		.07
Nonperseverative errors	1.49 (1.05)	.16	5.86 (1.01)	<.001	1.45 (1.06)		.17
Trials to first category	2.24 (1.39)	.11	6.32 (1.36)	<.001	2.92 (1.39)		.04
Matrices test^a^							
Abstract reasoning	−1.02 (0.65)	.12	−2.99 (0.63)	<.001	−0.42 (0.65)		.52

^a^ Analysis controls for age.

### Dimension vs Diagnosis

When the compulsivity factor and OCD diagnosis were present in the same model, the association between OCD diagnosis and model-based deficits approached zero (β [SE], −0.002 [0.02]; *P* = .91), whereas the association with compulsivity remained significant (β [SE], −0.05 [0.02]; *P* = .007) (Figure 3C). Thus, in a diagnosed patient population, scores on a self-reported compulsivity factor have more relevance to model-based deficits than diagnosis. Similar patterns were observed when diagnosis was compared with the compulsivity dimension for other cognitive measures. For cognitive flexibility, the number of categories completed (compulsivity: β [SE], −0.65 [0.10]; *P*<.001; OCD: β [SE], 0.18 [0.10]; *P* = .08), nonperseverative errors (compulsivity: β [SE], 6.37 [1.10]; *P*<.001; OCD: β [SE], −1.25 [1.11]; *P* = .26), and trials to first category (compulsivity: β [SE], 6.50 [1.49]; *P*<.001; OCD: β [SE], −0.45 [1.50]; *P* = .76) were each associated with the compulsivity factor and not OCD. Perseverative errors were not significantly associated with the compulsivity factor (β [SE], 1.12 [0.65]; *P* = .08) or OCD diagnosis (β [SE], −0.61 [0.65]; *P* = .35). Finally, abstract reasoning was associated with the compulsivity factor (β [SE], −3.79 [0.69]; *P*<.001), but OCD diagnosis was surprisingly associated with improved performance after controlling for compulsivity (β [SE], 1.95 [0.69]; *P* = .005). All results were maintained following Bonferroni correction for multiple comparisons unless otherwise stated.

### Replicability

We tested whether the associations between symptom dimensions and cognition could be replicated in a smaller set of individuals from whom we collected follow-up data (n = 110). We used the factor loadings defined at time 1 to define scores on the dimensions at time 2. We tested a priori hypotheses based on the results from testing session 1 and as such did not apply an additional correction for multiple comparisons. All associations between compulsivity and cognition were replicated: goal-directed control (β [SE], −0.04 [0.02]; *P* = .04), number of categories completed on the WCST (β [SE], −0.31 [0.14]; *P* = .02), nonperseverative errors (β [SE], 4.82 [1.51]; *P* = .002), trials to complete first category (β [SE], 5.95 [2.04]; *P* = .004), and abstract reasoning (β [SE], −3.60 [0.97]; *P* < .001). Consistent with the baseline testing session, there was no significant association with compulsivity and perseverative errors (β [SE], 0.92 [0.71]; *P* = .20). Obsessionality was associated with none of these cognitive assessments (model based: β [SE], −0.03 [0.02]; *P* = .16; number of categories: β [SE], −0.21 [0.14]; *P* = .13; perseverative errors: β [SE], 0.10 [0.72]; *P* = .89; nonperseverative errors: β [SE], 1.64 [1.57]; *P* = .30; trials to first category: β [SE], 3.31 [2.09]; *P* = .12; and abstract reasoning: β [SE], −1.62 [1.02]; *P* = .11). General distress was likewise not associated with model-based planning (β [SE], −0.02 [0.02]; *P* = .22), perseverative errors (β [SE], 0.70 [0.71]; *P* = .33), nonperseverative errors (β [SE], 1.73 [1.57]; *P* = .27), or trials to first category (β [SE], 2.34 [2.10]; *P* = .27) but was associated with completing fewer categories on the WCST (β [SE], −0.31 [0.14]; *P* = .02) and poorer performance at on the test of abstract reasoning (β [SE], −2.01 [1.01]; *P* = .05). These latter 2 results are considered unreliable, as they were not observed at time 1 (general distress and categories completed at time 1: β [SE], −0.11 [0.10]; *P* = .26; general distress and abstract reasoning at time 1: β [SE], −1.02 [0.65]; *P* = .12).

## Discussion

In this internet-based study of cognition with 285 patients diagnosed with OCD, GAD, or both, we found that having an OCD diagnosis was not associated with a reduction in goal-directed control. In contrast, a significant and replicable association was observed between self-reported scores on a compulsivity dimension (which manifested transdiagnostically) and deficits in goal-directed planning. When OCD diagnosis and compulsivity were included in the same analysis, the effect of OCD on goal-directed planning approached 0. Other symptom dimensions were identified in this study, corresponding to obsessionality and general distress factors. These variables had no robust association with goal-directed deficits.

Goal-directed deficits in patients with OCD have been previously described compared with healthy volunteers.^22,23,24,25^ However, no prior study, to our knowledge, has compared patients with OCD with a psychiatric control group in which deficits would not be anticipated, such as GAD. We found that an OCD diagnosis may not best capture these deficits, in contrast to a compulsivity factor, which can be expressed transdiagnostically in the general population^17^ and in patients with mental health disorders. The finding that goal-directed deficits were specific to the compulsivity dimension (vs general distress) aligns with the recent suggestion that trait anxiety^17^ and major life stress^17^ have no association with planning failures. Although some studies^50,51,52^ have found that acute stressors impair goal-directed planning, others^53,54,55^ have failed to replicate this, and acute anxiety induction appears to have no association with goal-directed control.^56^ Beyond general distress, these data suggest that obsessionality is phenomenologically and neurobiologically distinct from compulsivity. Formalizing the distinction between these dimensions provides a new opportunity for research to more precisely characterize their distinct cognitive underpinnings in patients, in the general population, and across species.

These findings were not specific to model-based planning; we obtained similar findings in relation to cognitive flexibility assessed using the WCST^43^ and abstract reasoning using a task based on the Raven matrices.^17^ Performance of both tasks is reliably impaired in OCD,^37,39^ and abstract reasoning has been previously reported to correlate with model-based planning.^17^ Although OCD diagnostic status was not associated with any of the performance measures, compulsivity was associated with abstract reasoning and all but 1 measure of flexibility on the WCST. No reliable associations were found with obsessionality or general distress. These data corroborate the finding that the compulsivity dimension maps onto putatively underlying neurocognitive deficits more closely than diagnosis. It is a limitation that we did not include tests that were unrelated to compulsivity, but previous internet-based work has already found that compulsivity has a distinct pattern of cognitive impairment to anxious-depression^15^ and social withdrawal.^16^ A direction for future research may be to test the extent to which compulsivity, obsessionality, and general distress map dissociable cognitive processes in patients with diagnosed disorders.

This is not the first study to assess alternative formulations of psychiatric phenotypes. Examples of data-driven work include the identification of clusters that cut across disorders and show differential cognitive or neural correlates^57^ or biotypes within a depression sample based on resting state connectivity.^58^Although this approach has potential, absent of theory, the risk of overfitting is high.^59^ Our study adds to this literature by taking a theory-driven approach that focuses on goal-directed control, a neural process that has been examined across species,^18,60^ in the context of a rodent model of addiction,^27^ in OCD,^21^ and more recently in the context of a broader class of compulsive behaviors.^61^

With the exception of the diagnostic interview, all data were collected online. The ability to derive robust and replicable findings of clinical importance using this method has broad implications for increasing the scalability of research in psychiatry.^62^ Large samples are needed to move beyond cross-sectional, case-control designs and harness the complexity of individual experiences of mental health, particularly if these insights are to be leveraged into individual-participant level estimations.^58,63,64^ Internet-based studies assessing cognition and self-report symptoms are in most cases considerably faster and less expensive than in-person studies. As such, the demonstrated validity of this method paves the way for a new wave of online psychiatry research.

There are several implications of these findings for research and practice. If dimensions are more proximal to underlying biological states, the use of transdiagnostic dimensions might reduce noise in research studies that aim to identify biomarkers of phenotype or treatment response. If these results are confirmed and extended, these data suggest that a move toward dimensional stratification of patients in the clinic may be feasible and provide a pathway to enhanced care through individualized treatment assignment, for example.^58^ The replicability of the internet-based method for cognitive research in psychiatry makes large-scale psychiatry studies more achievable outside large centers and consortia, allowing for cheaper, faster, and better powered studies that incorporate replication as standard.

### Limitations

This study has limitations. Diagnosis was determined using a telephone-based structured clinical interview. Few studies have assessed differences between telephone-based and in-person clinical interview, but results thus far are promising.^65^ Recruitment was entirely internet based; whether this might affect the generalizability of our findings to treatment-seeking populations is unclear. Our definition of compulsivity was narrow because we constrained our investigation to OCD- and GAD-relevant symptoms. Prior work^17^ in the general population found that a broader definition of compulsivity (including aspects of addiction and eating disorders) showed a stronger association with goal-directed planning than OCD severity, suggesting that our results might have been bolstered by a broader definition of compulsivity. The compulsivity dimension is not intended to be definitive but to test comparative questions about dimensions vs diagnosis in a mixed OCD and GAD sample. Working toward a definition, an ideal study would measure a broader range of symptoms and recruit a large all-comers sample with representation from multiple compulsive and noncompulsive disorders, healthy controls, and others.

## Conclusions

The findings suggest that deficits in goal-directed planning in OCD may be more strongly associated with a compulsivity dimension than with OCD diagnosis. This result may have implications for basic research that aims to assess the association between brain mechanisms and clinical manifestations and for understanding the structure of mental illness.

## Notes

Supplement. **eMethods.** Supplemental Methods **eResults.** Supplemental Results **eTable 1.** Associations Between Original Questionnaires and Measures of Higher-Order Cognition **eTable 2.** Reproducibility: Association Between Transdiagnostic Dimensions and Key Task Performance Measures at Follow-up, Time 2 (N = 110) **eTable 3.** Computational Model Parameter Estimates at Time 1 (N = 285) **eTable 4.** Computational Model Parameter Estimates at Time 2 (N = 110) **eTable 5.** Results Associated With Additional Two-Step Task Performance Metrics Ascertained From the Regression and Computational Modeling Approaches at Time 2 (N = 285) And Follow-up, Time 2 (N = 110) **eFigure 1.** Percentage of the Sample Meeting Criteria for Each *DSM* Disorder Investigated in This Study **eFigure 2.** Results Derived From 1000 Permutations of Subsampling to Match Groups for Age
