# Supplementary material for: Comparison of the Association Between Goal-Directed Planning and Self-reported Compulsivity vs Obsessive-Compulsive Disorder Diagnosis
Source: JAMA Psychiatry. 2019 Oct 9;77(1):77–85. doi: 10.1001/jamapsychiatry.2019.2998 (PMC6802255; doi:10.1001/jamapsychiatry.2019.2998)
Supplement: Supplement. — eMethods. Supplemental Methods eResults. Supplemental Results eTable 1. Associations Between Original Questionnaires and Measures of Higher-Order Cognition eTable 2. Reproducibility: Association Between Transdiagnostic Dimensions and Key Task Performance Measures at Follow-up, Time 2 (N = 110) eTable 3. Computational Model Parameter Estimates at Time 1 (N = 285) eTable 4. Computational Model Parameter Estimates at Time 2 (N = 110) eTable 5. Results Associated With Additional Two-Step Task Performance Metrics Ascertained From the Regression and Computational Modeling Approaches at Time 2 (N = 285) And Follow-up, Time 2 (N = 110) eFigure 1. Percentage of the Sample Meeting Criteria for Each DSM Disorder Investigated in This Study eFigure 2. Results Derived From 1000 Permutations of Subsampling to Match Groups for Age [file jamapsychiatry-77-77-s001.pdf]

Supplementary Methods

Gillan CM, Kalanthroff E, Evans M, et al. Comparison of the association between goal-directed planning and self-reported compulsivity vs obsessive-compulsive disorder diagnosis. *JAMA Psychiatry.* Published online October 9, 2019. doi:10.1001/jamapsychiatry.2019.2998

eMethods. Supplemental Methods

eResults. Supplemental Results

eTable 1. Associations Between Original Questionnaires and Measures of Higher-Order Cognition

eTable 2. Reproducibility: Association Between Transdiagnostic Dimensions and Key Task Performance Measures at Follow-up, Time 2 (N = 110)

eTable 3. Computational Model Parameter Estimates at Time 1 (N = 285)

eTable 4. Computational Model Parameter Estimates at Time 2 (N = 110)

eTable 5. Results Associated With Additional Two-Step Task Performance Metrics Ascertained From the Regression and Computational Modelling Approaches at Time 2 (N = 285) And Follow-up, Time 2 (N = 110)

eFigure 1. Percentage of the Sample Meeting Criteria for Each *DSM* Disorder Investigated in This Study

eFigure 2. Results Derived From 1000 Permutations of Subsampling to Match Groups for Age

This supplementary material has been provided by the authors to give readers additional information about their work.

Recruitment, Screening and Diagnostic Interview. Internet-based advertising was conducted via support groups, informational websites and mailing lists pertaining to OCD and anxiety, in addition to more general advertising via Craiglist and other online forums. After clicking on our advertisement, reading the study information and providing consent, subjects then supplied their age, email address, and answered some questions relevant to their eligibility, including self-reported symptoms of OCD and GAD using the DSM-5 criteria. 1136 individuals provided self-report data suggestive of a diagnosis of OCD or GAD, a valid email address, indicated that English was their first language, and had no history of stroke, serious head injury or neurological problems. These individuals were invited via email to proceed to a more extensive diagnostic interview via telephone, using an electronic version of the Mini-International Neuropsychiatric Interview (MINI 7.0) for DSM-5. To proceed, volunteers were required to make contact with a diagnostic screening site and arrange a time for their phone interview, as per the instructions in their invitation email. Subjects were allocated a diagnostic interview site based on scheduling constraints, mostly the availability of diagnostic interviewers at that site on a given week. The vast majority of potential participants never followed-up and were not contacted again. We interviewed 394 potential participants with whom we were able to schedule phone interviews. Participants were excluded following interview if they did not meet criteria for either OCD or GAD. Following interview, 335 individuals met criteria, 43 subjects were deemed ineligible because they did not meet criteria for a diagnosis of OCD or GAD and a further 16 withdrew. Those patients who met criteria were sent a link by email to participate in the main portion of the study (cognitive tests and clinical self-report). Of the 335 invited to participate in the study, 285 completed all parts of the study.

Diagnostic interviewer credentials. Diagnostic interviewers were based at 5 sites in the United States (New York State Psychiatric Institute, Massachusetts General Hospital, Butler Hospital, Zucker Hillside Hospital and New York University) and each had a minimum of a Master's degree in a clinical field, had previously done structured clinical evaluations on > 25 patients with a diagnosis of OCD and if not a licensed clinician, were trained in the conduct of the MINI diagnostic interview by a supervising clinician.

Patient Comorbidity. Subjects met criteria for on average, 3.7 DSM diagnoses of the 19 investigated (SD=2, range 1-9). The diagnoses assessed using the MINI were OCD, GAD, Major Depression, Social Phobia, Agoraphobia, Panic Disorder, PTSD, Substance Use Disorder (coded present if met criteria for any substance use disorder, including alcohol), Bulimia, Anorexia, Binge-Eating Disorder, Psychotic Disorders, and Bipolar I and II. This with custom DSM- based diagnostic interview questions pertaining to disorders not included in the MINI, which were Trichotillomania, Body Dysmorphic Disorder, Excoriation, Hoarding Disorder, Obsessive-Compulsive Personality Disorder (OCPD). After OCD and GAD, which were selected for in this study, OCPD, Social Phobia and Major Depression were the next most frequent diagnoses, present in 55.8%, 33% and 31% of the sample, respectively (eFigure 1). On the rarer end, Anorexia (2 cases), Psychotic Disorder (3 cases), Bipolar I (3 cases) and II (0 cases) were identified in <=1% of the population. Comparing the subsets who had OCD-only versus those with GAD-only, comorbidity profiles were extremely similar; only Major Depression differed in incidence between these subgroups, with GAD-only patients having more cases than OCD-only (Chi Sq=2.8, *p*=04).

© 2019 Gillan CM et al. *JAMA Psychiatry.*

eFigure 1. Percentage of the sample meeting criteria for each DSM disorder investigated in this study.

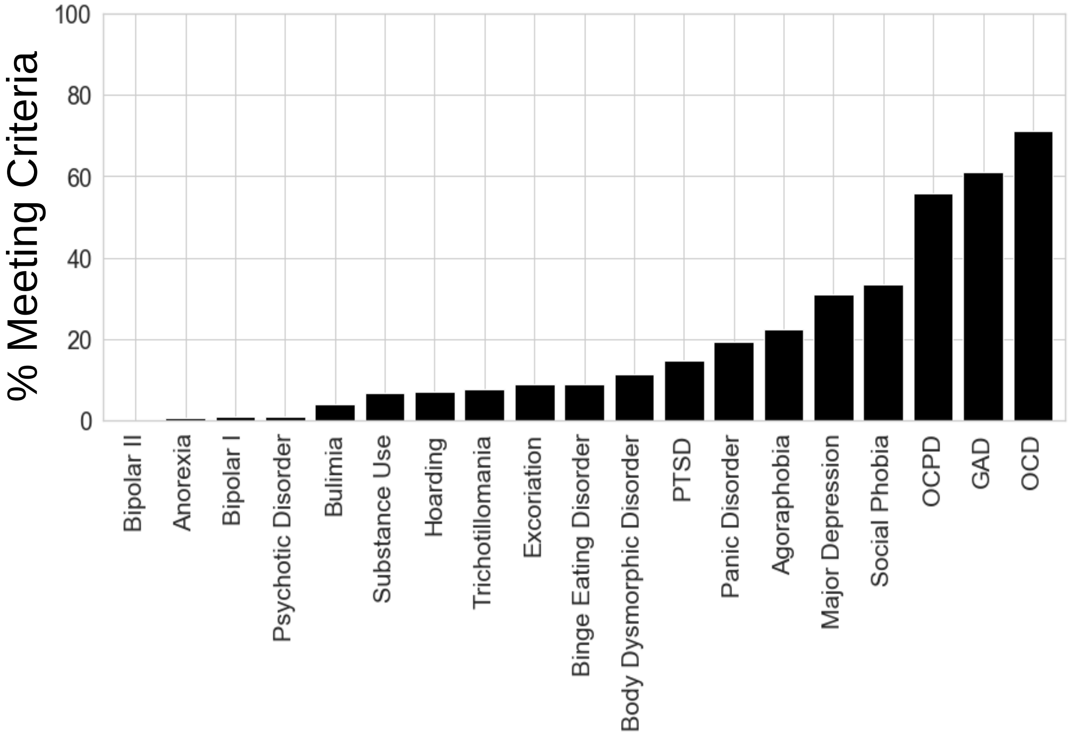

Model-based planning: hierarchical derivation of key parameters. To analyze two-step data, we first carried out a hierarchical regression analysis in R, that captures the extent to which subjects are likely to repeat an action that has been rewarded on the previous trial, in a manner dependent on the transition structure of the task (i.e., whether that action was followed by a rare or common transition). We extracted parameter estimates (regression coefficients) for model-based planning for each subject. These scores were then brought forward to simple linear regression models where age was controlled for and psychiatric variables of interest included. Analyses were repeated using a more elaborate computational model that allows characterization of potentially informative decision-making parameters (learning rate, choice stochasticity, model-free learning, perseveration) that were not the topic of specific hypotheses in this paper. These results are presented in detail in the supplementary results below. We did not exclude participants based on performance characteristics to avoid the prospect that we might remove meaningful behavior (e.g. rigidity) and because prior work that showed no advantage of fit to clinical variables from imposing quality control restrictions that reduce overall sample size[17].

WCST task and analysis. In the WCST, participants were presented the same four cards at the top of the screen on every trial and were asked to select which of these cards (or “decks”) they would like to sort a target card, which was presented on the bottom of the screen. The consistent presentation of the top cards allowed individuals to sort the target card on the basis of color (red, green, blue, yellow), shape (circle,

© 2019 Gillan CM et al. *JAMA Psychiatry.*

star, square or cross) or number of stimuli (1,2,3,4). Subjects were told that the correct answer depends on a rule, but that they would not be told what the rule was. If subjects sorted the card into the correct deck, they received “CORRECT” feedback, but if they sorted it into the wrong pile, they were told they were “WRONG”. Once subjects had made 10 consecutive correct selections, the rule changed so that another feature became the correct matching criteria. Subjects were warned that this would happen but were not informed of the specific conditions under which the rule would change. The task continued until they successfully completed 6 sets of individual rules or until 128 trials had been completed. Cognitive flexibility was operationalized in the standard way, using the number of categories completed and the number of perseverative errors made (cards sorted according to the previous rule) as the key measures. Additional correlated measures are also reported, including trials to first category, and the number of non-perseverative errors.

Abstract reasoning task and analysis. Abstract reasoning ability was assessed using a task based on Raven’s Progressive Matrices[41]. This test was adaptive, such that items became progressively more difficult when answered correctly and progressively easier when answered incorrectly. Items were selected using Maximum Fisher Information criterion[42] with a randomesque parameter of n=3[43] and the scores were estimated using a Bayes Modal estimator[44].

Factor analysis. Transdiagnostic dimensions were derived using an exploratory factor analytic method using the fa() function from the Psych package in R, with an oblique rotation (oblimin) and maximum likelihood estimation. The 15 subscales from the 4 questionnaires were entered as measured variables into the factor analysis, achieving close to a 20:1 ratio of subjects to variables. The number of factors to retain was selected based on an objective implementation of Cattell’s criterion (Cattell, 1966); wherein a sharp transition from horizontal to vertical (‘elbow”) indicates that there is little benefit to retaining additional factors. This was implemented using the Cattell-Nelson-Gorsuch test, which computes the slopes of all possible sets of three adjacent eigenvalues and determines the greatest differences in slope (nFactors package in R). The CNG test indicated a 3-factor structure, which comprised factors that we labelled “Obsessionality,” “Compulsivity” and “General Distress” based on inspection of loadings. The method employed was identical to that in the prior publication[17] with a different set of variables, which was later replicated[15] and shown to explain new data[14]. The compulsivity factor was related to OCD diagnostic status but were far from colinear. Specifically, Compulsivity accounted for just 18% of the variance in OCD diagnosis.

eResults

Self-Report Psychiatric Symptoms in their Original Form. Self-report questionnaire data in the original form were extremely similar to the dimensions presented in the main paper, and as such showed several significant, albeit less-specific, associations with goal-directed (model-based) performance. Total scores on the OCI-R: β=-0.03(0.02), p=.04, DASS: β=-0.04(0.02), p=.006, and global functional impairment on the Sheehan disability scale: β=-0.03(0.02), p=.03 were all linked to poorer model-based planning. Higher scores on the MCQ showed no significant association with model-based planning: β=-.02(.02), *p*=.17, but trended in the same

© 2019 Gillan CM et al. *JAMA Psychiatry.*

direction as each of the other questionnaires. Detailed results for all measures are presented in eTable 1.

eTable 1. Associations between original questionnaires and measures of higher-order cognition.

	OCI-R	DASS	MCQ	Sheehan
**Two-Step**				
Model-Based	**-0.03(0.02), p=.04**	**-0.04(0.02), p=.006**	-0.02(0.02), p=.17	**-.03(0.02), p=03**
**WCST**				
N Cat. Complete	**-.54(0.09), p<.001**	-0.09(0.10), p=.35	-0.13(0.10), p=.20	**-0.22(.10), p=.03**
Persev. Err	0.62(0.59), p=.29	0.07(0.58), p=.90	-1.05(0.58), p=.07	0.03(0.59), p=.96
Non-Persev. Err	**5.57(1.01), p<.001**	1.31(1.05), p=.21	1.93(1.05), p=.07	**2.17(1.05), p=.04**
Trials to 1st Cat.	**6.32(1.35), p<.001**	2.1(1.39), p=.13	**3.86(1.37), p=.005**	2.47(1.39), p=.08
**Matrices Test**				
Abstract Reasoning	**-2.58(0.64), p<.001**	-1.07(0.65), p=.10	-0.66(0.65), p=.31	-0.29(0.65), p=.66

eTable 2. Reproducibility. Association between trans-diagnostic dimensions and key task performance measures at follow-up, Time 2 (N=110).

	General Distress	Compulsivity	Obsessionality
**Two-Step**			
Model-Based	-0.02(0.02), p=.22	**-0.04(0.02), p=.04***	-0.03(0.02), p=.16
**WCST**			
N Cat. Complete	**-.31(0.14), p=.02***	**-.31(0.14), p=.02****	-0.21(0.14), p=.13
Persev. Err	0.70(0.71), p=.33	0.92(0.71), p=.20	0.10(0.72), p=.89
Non-Persev. Err	1.73(1.57), p=.27	**4.82(1.51), p=.002****	1.64(1.57), p=0.30
Trials to 1st Cat.	2.34(2.10), p=.27	**5.95(2.04), p=.004****	3.31(2.09), p=0.12
**Matrices Test**			
Abstract Reasoning	**-2.01(1.01), p=.05***	**-3.60(0.97), p<.001*****	-1.63(1.02), p=.11

Comparison of factors to original questionnaires

The intention behind searching for factors instead of using the original questionnaires was to allow separation of obsessions from compulsions, to test which of these dimensions explains the link between model-based planning and OCD symptomatology. The resulting factors achieved this, but were necessarily highly correlated with total scores on the original questionnaires. The correlation between the DASS and the general distress factor was r=.98, OCI-R and the compulsivity factor was r=.96; and MCQ and the obsessionality factor as r=.91. Despite being minor, there was evidence that the reformulation suggested by the factor analysis was more appropriate. For example, individual analyses on each of the 6 subscales of the OCI-R revealed that the 5 subscales that contribute most to the compulsivity factor all show associations with model-based planning in negative direction (checking, β = -.05(0.02), p=.001**; neutralising, β = -0.04(0.02), p=.005**; ordering, β = -.02(0.02), p=.07; washing, β = -.02 (0.02), p=.12; hoarding, β = -.005(0.02), p=.73), while the obsessions subscale, which loaded with the MCQ to form an obsessionality factor, trended in the opposite direction (obsessions β =.013 (0.02), p=.39). Similarly for abstract reasoning, the 5 subscales that contributed most to compulsivity all show

© 2019 Gillan CM et al. *JAMA Psychiatry.*

associations in a negative direction (checking, β = -3.17, p<.001**; neutralising, β = -2.38(0.64), p<.001***; ordering, β = -1.60(0.65), p=.015*; washing, β = -2.53(0.63), p<.001***; hoarding, β = -.1.38(0.65), p=.03*). while the obsessions subscale has no relationship, β = -.01(0.65), p=.99**.**

It follows then that despite their high correlation, when OCI-R and Compulsivity are included in the same model predicting model-based planning, Compulsivity shows a strong negative association (p<.001***), while the residual on OCI-R (which is essentially he obsession subscale) actually predicts enhanced model-based performance (p=.007**). In the same model that swaps model-based planning for abstract reasoning, compulsivity is again significant (p=.005**), while the residual on the OCI-R shows a trend in the opposite direction (p=.14).

Medication Effects. More than half of the patients included in this paper were medicated during the baseline testing session. Although medication status did not differ systematically across diagnostic groups, we found that those who were medicated had lower scores on the compulsivity dimension, β=0.28 (.12), p=.02 and had enhanced performance at model-based planning, β=.08(.03), p=.01, which presented a potential confound to interpretation. A regression analysis with both medication and compulsivity included as predictors revealed that these effects were independently significant (medication on model-based: *p*=.01; Compulsivity factor on model-based: *p*=.008).

Comorbidity. We complemented our analysis of the impact of the General Distress factor on cognition by testing if the number of diagnoses met (of all 19 disorders assessed in the structured clinical interview) had an influence on our key measures of higher order cognition. Much like the general distress factor, we found that having a greater number of diagnoses was marginally associated with model-based deficits (*p*=.06). This effect was no longer significant (p>.38) when the compulsivity factor was accounted for, adding weight to the broad finding that these deficits have a quite specific clinical manifestation in compulsivity.

Subsample analyses (age). Both OCD diagnosis and Compulsivity were associated with older age, which is linked to reduced model-based planning. Although age was controlled for in all analyses and the extent of the variance in OCD and compulsivity captured by age is relatively small (3% and 4% respectively), we nonetheless carried out an additional control analysis to eliminate the possibility that age differences across groups were responsible for our results. Specifically, we used propensity score matching using the MatchIt package in R to subsample our dataset down from 285 to 162, providing a 1:1 ratio of OCD+ to OCD– cases (82 GAD, 41 OCD, 41 OCD+GAD), with no remaining association between OCD and age (p=.93). Because there are numerous age-matched sub-samples that can be extracted from this dataset, we carried out 1000 iterations of propensity score matching and report results averaged across these 1000 permutations. Across 1000 age-matched subsamples, we found that the association with compulsivity and model-based planning was on average equivalent to that obtained in the full sample beta = -.05 (eFigure 2). Although statistical power was reduced through this subsampling procedure, the results were nonetheless significant in these subsamples, averaging p=.04. There was no association between OCD and model-based planning (beta=-.02, p=.39) in these age-matched subsamples, corroborating the main finding of this paper in the full sample.

© 2019 Gillan CM et al. *JAMA Psychiatry.*

Subsample analyses (medication). For information, we also broke down our sample into even smaller subgroups of medicated and unmedicated patients and repeated our key analyses relating cognitive test measures to compulsivity and OCD diagnosis. Results from the main analysis, where 5/6 tests showed an association with compulsivity, were consistent with that from the subgroups. Impaired performance on 5/6 cognitive tests was significantly associated with compulsivity in medicated individuals (N=157) and 4/6 in the unmedicated subgroup (N=126). OCD diagnosis was not linked to deficits in performance relative to the GAD baseline in any test, in either medicated or unmedicated groups. The only substantive difference across medication-status sub-samples was that the association between compulsivity and model-based planning was only evident in the medicated subgroup (p=.002), but not the unmedicated subgroup (p=.61). Given that this was not true of the other 5/6 tests, that we face a substantial loss of statistical power in these subsamples, that this medicated/unmedicated difference arose from 24 additional unplanned tests, and that if this effect was robust, there would remain a substantial interpretive difficulty associated with non-randomised medication-status effects, we caution against drawing a firm conclusion from this analysis.

Subsample analyses (disorder category). Finally, we tested if associations between goal-directed control and other cognitive measures were qualitatively consistent across individual disorder groupings, as a way to test the extent to which these findings are truly transdiagnostic in nature. The direction of effects were consistent across subsamples and the magnitudes broadly comparable. For example, the effect of compulsivity on model-based planning in the OCD only group was β =-.04(.02), p=.10; similar to the GAD-only subsample, β=-.03(.03), p=.31 and OCD+GAD subsample, β=-.06 (.03), p=.02. The association between abstract reasoning and compulsivity in the OCD-only group (β =-4.59(.96), p<.001) was almost identical to the GAD-only subsample (β =-.3.30(1.19), p=.007), but qualitatively larger than the OCD+GAD subsample, β =-2.01 (1.4), p=.08. Results on the WCST tended to be smaller in the GAD-only group, but again consistent in direction. The association between compulsivity and the number of categories completed was β =-.73(.16), p<.001 and β =-.62(.15), p<.001 for OCD-only and OCD+GAD, respectively, and β =-.23(.27), p=.17 for GAD-only. The association between compulsivity and non-perseverative errors was β =6.62 (1.96), p=.001 and β =6.56(1.52), p<.001 for OCD-only and OCD+GAD, respectively, and β =2.9(1.51), p=.06 for GAD-only. Finally, the association between compulsivity and trials to 1st criterion was β =6.76(2.65), p=.01 and β =7.78(2.33), p=.001 for OCD-only and OCD+GAD, respectively, and β =1.58(1.59), p=.33 for GAD-only.

© 2019 Gillan CM et al. *JAMA Psychiatry.*

eFigure 2. Results derived from 1000 permutations of subsampling to match groups for age.

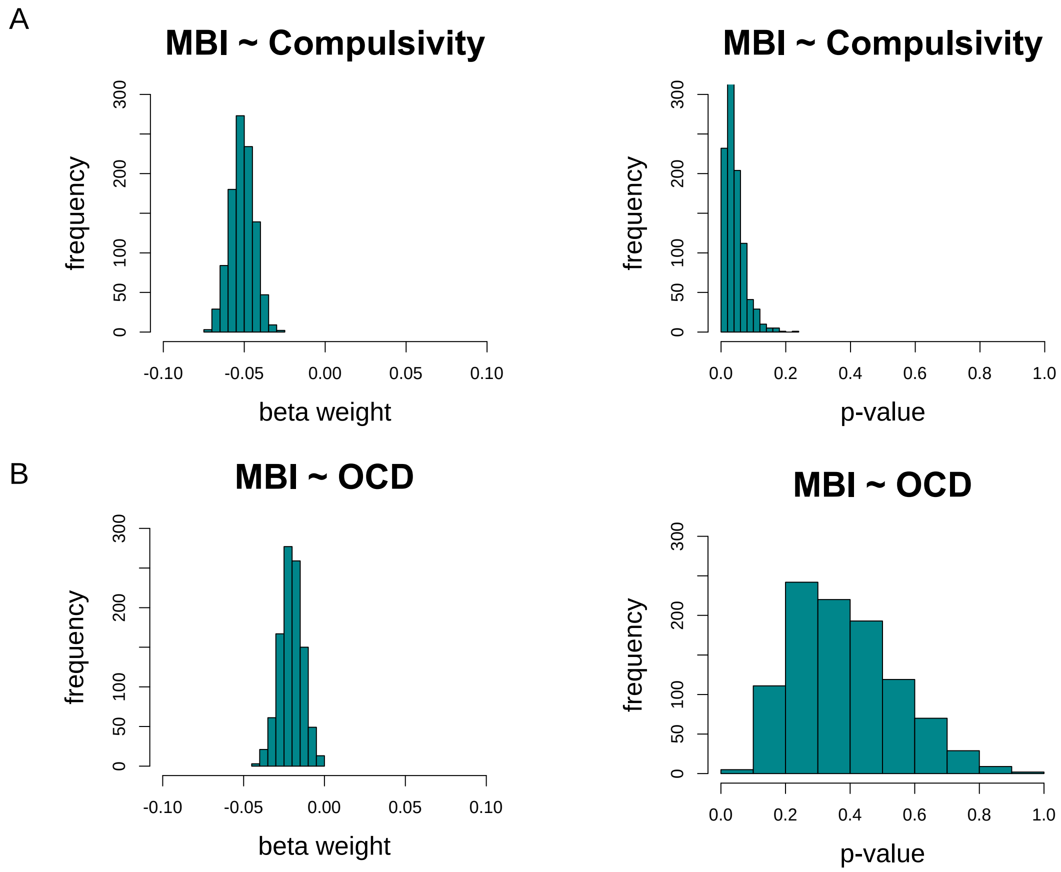

A. The effect of compulsivity on model-based planning was on average significant across 1000 permutations of smaller age-matched samples of N=164 subjects, mean beta=-.05 (left panel), mean p=.04 (right panel).
B. The effect of OCD on model-based planning was not significant in 1999 permutations, with an average beta of -.02 and p-value of .39.

Modeling of Two-Step Task. The simple regression-based analysis reported in the main text was complemented with a full computational model, which allows a characterization of not just model-based and model-free planning, but also choice stochasticity (choice randomness), perseveration and learning rate. Extensive detailing of the model employed can be found elsewhere [66]. In brief, the reinforcement-learning (RL) model used involves estimating a state-action value function for both model-based and model-free learning, which maps each possible action to its expected future reward. For the model-free algorithm, we used temporal difference (TD) learning [67], which is scaled by α the "learning rate" parameter. In effect, this measures the extent to which subjects are more likely to update their action preferences according to recent information (i.e., prediction error) versus the long-run estimates of value (i.e., Q values). The model-based RL algorithm works by learning the transition structure of the task and immediate reward values for each state, then computing cumulative state-action values by iterative expectation over these. To connect the values to choices, we used a softmax choice rule, where the inverse temperature parameter β captures the stochasticity of choices. Repetition was

© 2019 Gillan CM et al. *JAMA Psychiatry.*

measured and used to estimate a perseveration (repetition) parameter [68]. At both Time 1 and Time 2, all parameters were significant (eTables 3 and 4, respectively).

eTable 3. Computational Model Parameter Estimates at Time 1 (N=285)

**Parameter Estimates**	**Learning Rate**	**Repetition**	**Model-Based**	**Model-Free**	**Stochasticity**
**Upper 95%**	0.65	0.70	0.20	1.34	2.06
**Median**	0.61	0.59	0.14	1.21	1.89
**Lower 95%**	0.57	0.49	0.07	1.08	1.74

For each parameter, the median posterior estimate is given, together with the 95% confidence interval.

eFigure 4. Computational Model Parameter Estimates at Time 2 (N=110)

**Parameter Estimates**	**Learning Rate**	**Repetition**	**Model-Based**	**Model-Free**	**Stochasticity**
**Upper 95%**	0.70	0.92	0.21	1.69	2.29
**Median**	0.64	0.72	0.11	1.45	2.03
**Lower 95%**	0.59	0.53	0.01	1.21	1.78

For each parameter, the median posterior estimate is given, together with the 95% confidence interval.

Results from the computational model were largely consistent with that of the simpler one-trial-back regression (eTable 5). The compulsivity dimension was associated with model-based planning deficits at both time points, regardless of whether it was defined in the computational model or regression-based approach. We also found that choice repetition was decreased in those high in compulsivity, at both time points and in both the regression and computational model (T2 just marginal, p=.08, eTable 5).

To our surprise, we also observed an association between the compulsivity factor and model-free planning at Time 1 using both methods, such that greater compulsivity scores were linked to less model-free planning. This was not expected because several studies have previously investigated if model-free planning was linked to OCD diagnosis and/or symptoms, with none finding any evidence of an association[15,18,34,39]. As this effect did not persist at Time 2 using either method (regression: β=-.01(.05), *p*=.86; computational model: β=-.02(.10), *p*=.85), we attribute the un-hypothesized T1 finding to chance.

The computational model allows us to measure learning rate and choice stochasticity, which the regression cannot estimate. There were no significant associations with learning rate, but we did find that those high in compulsivity were higher in choice randomness at Time 1, a finding consistent with at least one prior study[15], but which did not reach significance at Time 2 (p=.13). OCD diagnosis was also associated with more stochasticity at Time 1 (p=.004). As diagnostic information was only available at Time 1, we could not check the consistency of this finding. Together with the finding of reduced repetition, this increase in stochasticity might

© 2019 Gillan CM et al. *JAMA Psychiatry.*

speak to the fact that individuals high in compulsivity are more difficult to fit behaviorally on this task – suggesting they may employ some other strategy not accounted for in the model.

eTable 5. Results associated with additional two-step task performance metrics ascertained from the regression and computational modelling approaches at Time 2 (N=285) and follow-up, Time 2 (N=110).

		OCD Diagnosis	General Distress	Compulsivity	Obsessionality
**Regression**	**Time**				
Model-Based	T1	-.02(.02), p=.18	**-.04(.02), p=.01***	**-.05(.02), p=.003****	-.01(.02), p=.63
	T2		-.02(.02), p=.23	**-.04(.02), p=.04***	-.03(.02), p=.16
Model-Free	T1	-.04(.03), p=.11	-.04(.03), p=.20	**-.09(.03), p<.001*****	-.02(.03), p=.52
	T2		-.08(.05), p=.08	-.01(.05), p=.86	-.04(.05), p=.41
Repetition	T1	-.09(.07), p=.18	-.10(.07), p=.16	**-.28(.07), p<.001*****	-.05(.07), p=.52
	T2		**-.26(.11), p=.02***	**-.22(.11), p=.05***	**-.30(.11), p=.007***
**Computational Model**	**Time**				
Model-Based	T1	-.02(.02), p=.38	**-.04(.02), p=.04***	**-.05(.02), p=0.02***	-.01(.02), p=.72
	T2		-.02(.02), p=.48	**-.05(.02), p=0.05***	-.02(.02), p=.46
Model-Free	T1	**-.11(.06), p=.05***	-.10(.06), p=.07	**-.23(.06), p<.001*****	-.06(.06), p=.30
	T2		**-.22(.10), p=.03***	-.02(.10), p=.85	-.15(.10), p=.12
Repetition	T1	-.02(.04), p=.59	.01(.04), p=.88	**.10(.04), p=0.03***	-.04(.04), p=.41
	T2		-.08(.09), p=.35	-.15(.08), p=0.08	-.14(.08), p=.11
Learning Rate	T1	-.02(.01), p=.16	-.01(.01), p=.33	-.03(.01), p=.07	-.01(.01), p=.57
	T2		-.001(.02), p=.96	.01(.02), p=.71	.03(.02), p=.18
Stochasticity	T1	**-.20(.07), p=.004****	**-.23(.07), p<.001*****	**-.40(.07), p<.001*****	-.10(.07), p=.16
	T2		-.08(.11), p=.42	-.16(.10), p=.13	-.02(.11), p=.82

Effects that were consistently observed in Time 1 (T1) and Time 2 (T2) are highlighted with a surrounding box. Significant effects are presented in bold, regardless of consistency across time points. All analyses control for age.

© 2019 Gillan CM et al. *JAMA Psychiatry.*
